# Patient-Centered Communication

**DOI:** 10.3390/pharmacy6010018

**Published:** 2018-02-13

**Authors:** Cynthia A. Naughton

**Affiliations:** School of Pharmacy, College of Health Professions, North Dakota State University Fargo, ND 58108, USA; Cynthia.Naughton@ndsu.edu; Tel.: +1-701-231-8487

**Keywords:** patient-centered communication, Calgary-Cambridge guide, four habits model, Patient-Centered Communication Tools (PaCT), communication models, pharmacists

## Abstract

As the population ages, morbidity and mortality associated with chronic disease will increase. Some patient-centered improvements have been made in health care services, but optimal health has not been fully realized. Only when pharmacists have a holistic understanding of an individual patient, including their experience of illness and medication, can they effectively assess appropriateness, safety, efficacy, and adherence to medications and develop realistic treatment plans. When patients are involved in their care, they are better able to manage complex chronic conditions by understanding and adhering to their plan of care. Pharmacists can enable patients to participate fully using patient-centered communication. There are relatively few published articles on patient-centered communication specific to pharmacists, but the Calgary-Cambridge guide and Four Habits model have applicability to pharmacy practice. The Patient-Centered Communication Tools (PaCT), created for use in pharmacy education and loosely based on the Four Habits model, can assist pharmacists in developing their patient-centered communication skills. Lastly, best practices for patient-centered communication in pharmacy practice are described.

## 1. Introduction

In 2007, the Institute for Healthcare Improvement launched its Triple Aim to focus on improving the patient’s experience of care, achieving better health outcomes, and reducing the per capita cost of health care [1]. A key reason for establishing the Triple Aim was that health care costs in the United States were skyrocketing without any apparent improvement in the overall health of its citizens [2]. A decade later, the United States continues to spend more on health care with poorer health care outcomes compared to 10 other developed countries [3]. Chronic disease claims 86% of the total annual expenditures for healthcare in the U.S. and accounts for seven of the top ten causes of death in American adults [4,5]. As the population ages, the burden of chronic disease morbidity and mortality will increase unless a more holistic approach to health is adopted.

Health, as defined by the World Health Organization, is “a state of complete physical, mental, and social well-being and not merely the absence of disease or infirmary” [6]. There are many factors that influence the state of a person’s health. Generally known as Determinants of Health, these factors can be grouped into five major categories (Table 1): (1) clinical health care; (2) genetic vulnerability; (3) socio-economic characteristics; (4) environmental and physical influences; and (5) individual health behaviors such as tobacco use, diet and exercise, and alcohol and drug use [7]. Only the first two determinates, clinical health care and genetic vulnerability, have a direct biological connection to our health. Socio-economic characteristics such as educational level, employment, income, marital status, and ethnicity along with environmental and physical influences such as place of residence, quality of air and water, buildings, spaces, and transportation are often referred to as the Social Determinants of Health. In short, the Social Determinants of Health are the conditions in which people are born, grow, live, work, and age [8]. The relative contribution of each health determinant towards overall health varies depending upon the disease, population, and geographical region. Typically, health care services and genetics only account for 10–20% of a person’s health, whereas the largest contribution comes from the Social Determinants of Health as well as individual health behavior [9,10]. Therefore, rather than investing more dollars into healthcare services, factors that play a greater role in health are deserving of attention [11,12].

**Figure 1:**
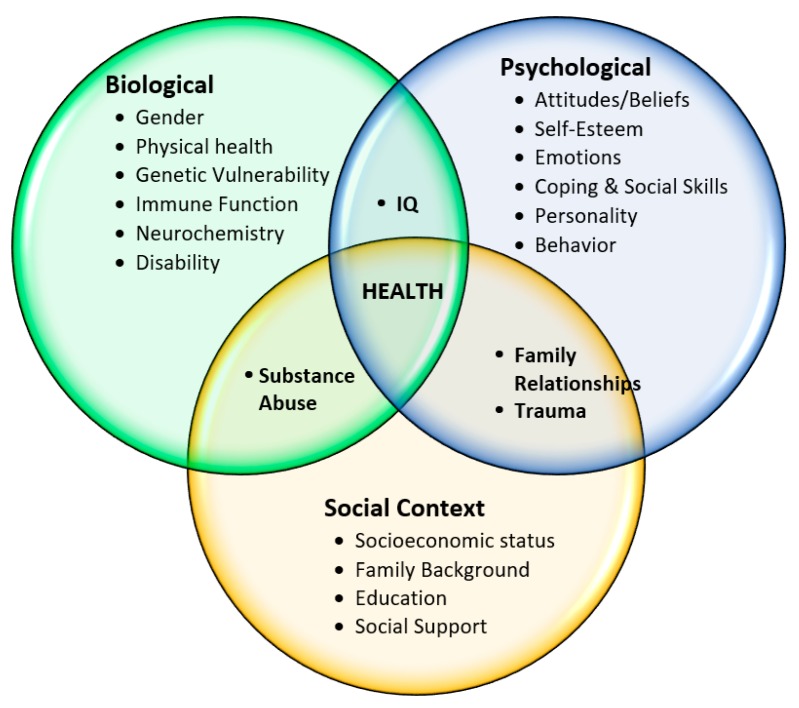
Biopsychosocial Model of Disease and Illness.

Fortunately, modern medicine is moving away from a purely biomedical model of care with an emphasis on disease and its associated biological components (signs, symptoms, and laboratory tests) to a biopsychosocial model. The biopsychosocial model of care is a holistic framework to describe and explain how illness is the result of the interplay of biological, psychological, and social factors plus individual health-related behaviors (Figure 1) [13]. Recognizing, understanding, and responding to all factors that affect health requires the healthcare provider to integrate the biological aspects of the disease with the psychological and social aspects of the patient. The goal of this model is to develop a patient-centered care plan that is realistic in order to achieve the best possible health outcomes.

Another major improvement made in health care services delivery was the adoption of patient-centered care. The Institute of Medicine defines patient-centered care as “*a partnership among practitioners, patients, and their families ensures that decisions respect patients’ wants, needs, and preferences, and that patients have the education and support they need to make decisions and participate in their own care, as well as participate in quality improvement efforts*” [14]. The term patient- and family-centered care acknowledges the importance of families on the health of patients of all ages in all settings of care as well as being essential allies for quality and safety [15]. In recent years, person-centered care has emerged as a new term that encompasses the entirety of a person’s needs and preferences (biopsychosocial) beyond just the pathophysiology of the disease (biomedical) [16]. While the practice of pharmacy employs medications as its primary means of health care intervention, the professional and ethical responsibility of pharmacists are clearly more holistic. Pharmacists, in their Code of Ethics, promise to place the “well-being of the patient at the center and consider their stated needs as well as those defined by science” [17].

When patients are more involved in their care, they are better able to manage complex chronic conditions by understanding and incorporating their plan of care, are more likely to feel comfortable communicating their concerns and seeking appropriate assistance, have reduced anxiety and stress, and have shorter lengths of stay [18]. Patients involved in their own care also have a safer care experience [19]. Therefore, all healthcare providers have a professional and ethical responsibility to encourage patients to express their concerns. The effectiveness of patient-provider communication is not always optimal, however. For instance, early studies on patient recall of medical information showed that 40–80% of medical information provided by healthcare practitioners is forgotten immediately and nearly half of what is remembered is remembered incorrectly [20]. It is not surprising then that patient adherence to treatment recommendations for chronic disease varies between 37 and 87%, and only 50% of all prescription drugs are taken as prescribed [21,22]. To enable patients to participate fully in their care, healthcare professionals need to facilitate optimal information exchange using patient-centered communication.

## 2. Patient-Centered Communication

The core concepts of patient-centered communication include “(1) eliciting and understanding patient perspectives (e.g., concerns, ideas, expectations, needs, feelings, and functioning), (2) understanding the patient within his or her unique psychosocial and cultural contexts, and (3) reaching a shared understanding of patient problems and the treatments that are concordant with patient values” [23]. Although health care providers acknowledge that patients should play a more participative role to ensure they are informed about their care, several barriers to communication exist.

The first barrier to patient-centered communication is a perceived lack of time. Practitioners may feel they lack enough time to listen, explain, and negotiate with the patient. Sometimes patients are not able to fully articulate their initial concerns before being interrupted by the provider. In a study involving physicians and agenda-setting with patients, patients were interrupted after an average of 23.1 s [24]. Studies show, however, that patients rarely take more than 2–3 min to share their whole story when asked open-ended questions and are not interrupted [25,26]. Shared decision-making also takes time but on average only an additional 10% of the entire duration, i.e., 2 min for a 20 min encounter [27].

The second barrier relates to negotiating evidence-based treatment plans with patients. Evidence found in the literature to support treatment is often “disease-oriented” with reference to lab values, plaque size, or blood pressure; all of which are markers for disease outcomes rather than actual outcomes. Most patients have no frame of reference for the impact of those numbers. “Patient-Oriented Evidence that Matters” (POEMs); on the other hand, refer to outcomes that patients care about and can relate to [28]. Examples of POEMs include outcomes related to morbidity (symptoms), daily functioning, mortality, cost, and quality of life as defined by the patient [29]. Another consideration is that evidence-based medicine corresponds to population data and may not reflect the needs and preferences of individual patients. Rather than the rule, evidence-based medicine should only be a guide used along with provider expertise and the patient’s goals, values, and preferences [30].

Finally, provider attitude can be a barrier to effective patient-centered communication. Traditionally, pharmacists have been educated as drug experts and have been taught about the pharmacologic and pharmacotherapeutic properties of a drug to meet a patient’s medication-related needs and promote medication compliance [31]. Scientific drug knowledge is clearly important, but a patient-centered approach requires knowledge of the patient and their individual experience of illness and medication. Only after incorporating a holistic understanding of the patient’s beliefs, attitudes, and behaviors towards health can pharmacists assess the appropriateness of indication, effectiveness, safety, and adherence to medications. In other words, “the pharmacist must maintain a high level of humility about their scientific knowledge so that the knowledge of the patient can be recognized” [31]. Using a consistent approach to patient-centered care specific to pharmacy is advocated to assist pharmacists in fulfilling their professional responsibilities to a patient. The Pharmacists Patient Care Process (PPCP), supported by 13 national pharmacy organizations and the Accreditation Council for Pharmacy Education, is a model to optimize patient health and medication outcomes [32,33].

## 3. Pharmacists and Patient-Centered Communication

A patient-centered approach to communication is to acknowledge the whole person, their personality, life history, and social structure in order to develop a shared understanding of the problem, the goals of treatment, and the barriers to that treatment and wellness. With the practice of pharmacy expanding beyond the traditional medication dispensing roles, pharmacists must become competent in patient-centered communication. Expectations for professional communication in the 2016 Accreditation Council for Pharmacy Education (ACPE) guidelines for the Doctor of Pharmacy degree are found in Standard 3 (Approach to Practice and Care) and Appendix 1 [33]. There are many published articles on patient-centered communication in healthcare, but relatively few are specific to pharmacy. While the majority of publications are oriented towards physicians, three excellent examples with applicability to pharmacy practice are detailed below.

The Calgary-Cambridge guide was developed for use in medical education to teach and assess patient-centered communication [34,35]. It is widely used in over 60% of medical schools in the U.K. and is the second most-used guide in North America for teaching and assessing professional communication [36]. The guide’s framework corresponds to the structured process of a medical interview (initiating the session, gathering information, physical examination, explanation and planning, and closing the session) and consists of 71 communication skills and behaviors [36,37]. Although lengthy, the authors of the guide meant for it to be comprehensive but modifiable depending upon the nature of the medical encounter. In a recent study, the applicability of the Calgary-Cambridge guide to assess pharmacist–patient communication was analyzed. Eleven pharmacists representing a variety of settings (e.g., community, primary care, and hospital) were observed and recorded during a total of 18 patient consultations. It was noted that many of the communication skills on the Calgary-Cambridge guide were represented during the pharmacist-led consultations and highlighted areas in which pharmacists may need more training [38].

The Four Habits Model is another framework for patient-centered communication also designed for physicians. It contains 23 clinician communication behaviors organized into four “habits”: invest in the beginning, elicit the patient’s perspective, demonstrate empathy, and invest in the end [39,40,41]. This model provides explicit examples of how to create rapport, elicit patient concerns and ideas, explore the illness experience, and convey empathy and can be helpful to other health care professionals wishing to improve their communication skills. The Four Habits Model was used as a foundation for the development of the Patient-Centered Communication Tools (PaCT) to measure pharmacy students’ communication skills [42].

The PaCT includes 23 clinical communication skills categorized into five “tools” (establish a connection, explore and integrate the patient’s perspective, demonstrate interest and empathy, collaborate and educate, and communicate with finesse). Each individual communication skill is scored using a five-point Likert scale (unsatisfactory, needs improvement, adequate, capable, and proficient). When comparing the PaCT and the Four Habits Model on the same performance, scores were significantly correlated. According to the authors, the instrument demonstrated significant face, content, construct, and test–retest validity [42].

## 4. Best Practices

Pharmacists provide patient care with varied responsibilities in a variety of practice settings. Pharmacist’s clinical expertise and access to patients, particularly in the retail setting, place them in a unique position to improve health outcomes of individual patients and populations alike. In many cases, retail pharmacies are the primary point of health care access in rural communities [43]. Regardless of practice setting, patient-centered communication such as openness, active listening, and plain speaking are three general skills in which all pharmacists should become competent.

### 4.1. Openness

Openness is demonstrated by making oneself available, not only with time but also by the manner in which the patient and their perspectives are acknowledged [31]. A curt greeting and appearing rushed or inconvenienced communicates to patients that their time and concerns are not important. Rather, identifying a patient by name in a warm greeting, offering a smile, being attentive, and maintaining friendly eye contact goes a long way in establishing rapport and building a relationship.

### 4.2. Active Listening

Attentive body language (e.g., open posture, eye contact, and interested expression), eliciting verbal (e.g., “uh-huh” and “I see”) and nonverbal (e.g., nodding) encouragement, paraphrasing to confirm understanding, and keeping questions to a minimum demonstrates to the patient a genuine interest in them on the part of the pharmacist. Questions designed to collect patient perspectives should be open-ended questions using the words “what” or “how” instead of those that can be answered with “yes” or “no.” Asking open-ended questions provide critical insight into the patient’s experience of illness, yield critical information to promote medical adherence, and facilitate shared decision making.

### 4.3. Speaking Plainly

Health literacy is the degree to which individuals have the capacity to obtain, process, and understand basic health information needed to make appropriate health decisions [44]. Only 12% of adults have proficient health literacy according to the National Assessment of Adult Literacy. In other words, 9 out of 10 adults may lack the skills needed to manage their health and prevent disease. It is helpful to consider all patients as having low heath literacy and use appropriate communication techniques that ensure understanding. At a minimum, slow down and speak in plain, non-medical language! Allow time for patients and families to ask questions by asking “What questions do you have?” instead of “Do you have any questions?” Check the understanding of a patient by asking them to restate it in their own words, not just repeat it, to ensure the message is understood.

Best practices of patient-centered communication in medical encounters have been gleaned from empiric evidence and patient satisfaction data [45]. The best practices are organized into six functions with corresponding communication skills for each function. Although the framework is geared towards the physician–patient relationship, many of the communication concepts are transferrable to patient encounters involving pharmacists (Table 2).

## 5. Conclusions

Pharmacists have a professional and ethical responsibility to consider the needs and situation of the patient holistically, in the psychological and social realms as well as the biological realm. Pharmacists can employ practical strategies to foster patient-centered communication that engages patients to participate in their care and facilitates in the development of a trusting pharmacist–patient relationship, leading to a shared understanding of the entire problem, the goals of treatment, and the barriers to wellness. Only then can a realistic plan of care be developed and followed, and in turn increase the likelihood of improved health outcomes.

## Figures and Tables

**Table 1:** Determinants of Health.

Determinants of Health [7]
clinical health care services
genetic vulnerability
socio-economic characteristics
physical environment
individual health behavior

**Table 2:** Best practices for Pharmacist Provided Patient-Centered Communication ^a^.

Goal	Pharmacist Responsibility	Communication Skills
Foster the Relationship	Build rapport Appear open Demonstrate respect Demonstrate caring and commitment Acknowledge feelings and emotions	Greet patient warmly and appropriately Maintain eye contact Show interest Listen actively Express empathy
Gather Information	Determine purpose of encounter Discover biomedical perspective (disease) Understand patient perspective (illness)	Ask open-ended questions Allow patient to complete responses Clarify and summarize information Explore impact of illness on patient
Provide Information	Identify patient informational needs Share information Overcome health literacy barriers	Speak plainly and avoid jargon Use “Patient-Oriented Evidence that Matters” (POEMs) Encourage questions Check for understanding
Share Decision-Making	Identify patient goals Outline collaborative treatment plan	Explore patient preferences Identify barriers to treatment choices Negotiate agreement
Enable Treatment Success	Assess the patient’s capacity for self-management Arrange for needed support Advocate for and assist patient with health system	Summarize treatment plan Elicit patient understanding Discuss follow-up

^a^ Adapted from King A, Hoppe RB. Best practice for patient-centered communication: A narrative review. JGME. 2013;5(3):385–393.
